# A PRISMA-compliant systematic review and meta-analysis of the relationship between thyroid disease and different levels of iodine intake in mainland China: Erratum

**DOI:** 10.1097/MD.0000000000009593

**Published:** 2018-01-05

In the article, “A PRISMA-compliant systematic review and meta-analysis of the relationship between thyroid disease and different levels of iodine intake in mainland China”,^[1]^ Jun Zhang's name appeared incorrectly.
